# Role of Human NADPH Quinone Oxidoreductase (NQO1) in Oxygen-Mediated Cellular Injury and Oxidative DNA Damage in Human Pulmonary Cells

**DOI:** 10.1155/2021/5544600

**Published:** 2021-10-15

**Authors:** Rebecca Burke, Chun Chu, Guo-Dong Zhou, Vasanta Putluri, Nagireddy Putluri, Rachel E. Stading, Xanthi Couroucli, Krithika Lingappan, Bhagavatula Moorthy

**Affiliations:** ^1^Section of Neonatology, Department of Pediatrics, Baylor College of Medicine, Texas Children's Hospital, Houston, TX, USA; ^2^Division of Neonatology, Department of Pediatrics, West Virginia University Morgantown, WV, USA; ^3^Institute of Biosciences and Technology, Texas A&M University Health Science Center, Houston, TX, USA; ^4^Advanced Technology Core, Baylor College of Medicine, Texas Children's Hospital, Houston, TX, USA; ^5^Department of Molecular & Cellular Biology, Baylor College of Medicine, Texas Children's Hospital, Houston, TX, USA

## Abstract

Supplemental oxygen administration is frequently used in premature infants and adults with pulmonary insufficiency. NADPH quinone oxidoreductase (NQO1) protects cells from oxidative injury by decreasing reactive oxygen species (ROS). In this investigation, we tested the hypothesis that overexpression of NQO1 in BEAS-2B cells will mitigate cell injury and oxidative DNA damage caused by hyperoxia and that A-1221C single nucleotide polymorphism (SNP) in the *NQO1* promoter would display altered susceptibility to hyperoxia-mediated toxicity. Using stable transfected BEAS-2B cells, we demonstrated that hyperoxia decreased cell viability in control cells (Ctr), but this effect was differentially mitigated in cells overexpressing NQO1 under the regulation of the CMV viral promoter, the wild-type *NQO1* promoter (*NQO1-NQO1*), or the *NQO1* promoter carrying the SNP. Interestingly, hyperoxia decreased the formation of bulky oxidative DNA adducts or 8-hydroxy-2′-deoxyguanosine (8-OHdG) in Ctr cells. qPCR studies showed that mRNA levels of *CYP1A1* and *NQO1* were inversely related to DNA adduct formation, suggesting the protective role of these enzymes against oxidative DNA injury. In SiRNA experiments entailing the *NQO1-NQO1* promoter, hyperoxia caused decreased cell viability, and this effect was potentiated in cells treated with *CYP1A1* siRNA. We also found that hyperoxia caused a marked induction of DNA repair genes *DDB2* and *XPC* in Ctr cells, supporting the idea that hyperoxia in part caused attenuation of bulky oxidative DNA lesions by enhancing nucleotide excision repair (NER) pathways. In summary, our data support a protective role for human NQO1 against oxygen-mediated toxicity and oxidative DNA lesions in human pulmonary cells, and protection against toxicity was partially lost in SNP cells. Moreover, we also demonstrate a novel protective role for CYP1A1 in the attenuation of oxidative cells and DNA injury. Future studies on the mechanisms of attenuation of oxidative injury by NQO1 should help in developing novel approaches for the prevention/treatment of ARDS in humans.

## 1. Introduction

Supplemental oxygen is an integral part of medical management of pediatric and adult patients with pulmonary insufficiency [1–3]. In premature infants and adults, exposure to hyperoxia contributes to the development of bronchopulmonary dysplasia (BPD) [4, 5], and in adults, it could exacerbate acute respiratory distress syndrome (ARDS) [6–8]. ARDS is a life-threatening illness that affects up to 10% of patients in intensive care units worldwide [9] and could develop following pneumonia, nonpulmonary sepsis, trauma, or aspiration [9]. Despite significant medical advances, mortality due to ARDS is high (35-46%) [8, 9], and recent studies have shown that ARDS is one of the major causes of death due to the COVID-19 infection [10]. The molecular mechanisms of oxygen-mediated lung injury are not completely understood, but reactive oxygen species (ROS) likely play an important role [11]. Hyperoxia (>95% FiO_2_) for 72 hours in rodents results in lung inflammation and injury, eventually leading to cell death [4, 12]. ROS generated in hyperoxic conditions lead to profound cell damage through direct DNA damage, lipid peroxidation, protein oxidation, and alteration of transcription factors [4, 12]. Recent studies from our laboratory have shown a protective effect of cytochrome P450 (CYP) 1A enzymes against hyperoxic lung injury *in vivo* [13–20].

NADPH quinone oxidoreductase 1 (NQO1) is a phase II enzyme whose activity in the cell is to catalyze the two-electron reduction of quinone compounds, which prevents the generation of ROS and, thus, protects cells from oxidative damage [21]. Das et al. showed that mice deficient in the genes for *Nqo1* and *Nqo2* are more susceptible to lung injury than wild-type mice [22]. A number of single nucleotide polymorphisms (SNPs) have been reported for NQO1 [23–28]. Although associations between genetic variants in *NQO1* and ALI/ARDS have been reported [22–28], little is known regarding the mechanisms by which these genetic variants contribute to ARDS.

Prior reports have demonstrated that the A/C single nucleotide polymorphism (SNP) at -1221 of the *NQO1* promoter resulted in attenuation of *in vitro* transcription of luciferase reporter expression following exposure to hyperoxic conditions [29]. Individuals in a cohort of trauma patients who were genotyped for the A-1221C SNP were found to have a significantly decreased incidence of acute lung injury (ALI), implying a protective role for A-1221C in ARDS patients [29].

The overall objective of this investigation was to study the role of human NQO1 and A-1221C SNP in hyperoxia-mediated cellular injury and oxidative DNA damage. Specifically, we tested the hypothesis that overexpression of NQO1 in BEAS-2B cells will mitigate cell injury and oxidative DNA damage caused by hyperoxia and that the presence of A-1221C SNP in the *NQO1* promoter would display altered susceptibility to hyperoxia-mediated toxicity.

## 2. Materials and Methods

### 2.1. Cell Culture

BEAS-2B adenovirus 12-SV40-transformed, normal human bronchial epithelial cells (ATCC) were maintained in RPMI 1640 medium supplemented with 10% FBS and penicillin-streptomycin at 37°C in room air containing 5% CO_2_. The hyperoxia condition used was 80% O_2_ plus 5% CO_2_.

### 2.2. Construction of Plasmids

A 2.4 kb of human *NQO1* promoter was obtained from the genomic DNA of BEAS-2B cells by the LA Taq PCR Kit (Takara) using primer pair GGCTTCTCAGACCACTCCTG and ACTAGGCTCTCGGTGAGCTG and subcloned into the pGL4.13 luciferase expression plasmid (Promega) between the SacI and XhoI sites. A-1221C mutation (rs689455) at the *NQO1* promoter region of the pGL4-NQO1 plasmid was introduced by site-directed mutagenesis PCR using primer pair AGGTCGGGAGTTGGAAAC and CAGGTGATCCTACCGCCT. These two plasmids were named pGL4-*NQO1* and pGL4-_SNP_*NQO1*.

To obtain the NQO1 expression plasmid pCD-*NQO1*, total RNA was extracted from BEAS-2B cells and subjected to reverse transcription using the SuperScript III First-Strand Synthesis System (Invitrogen). The open reading frame and the 3′-UTR of human *NQO1* were obtained as one piece by the subsequent PCR (Takara) using primer pair CAGCTCACCGAGAGCCTAGT and AAAAACCACCAGTGCCAGTC and then subcloned between the NheI and XhoI sites of the pcDNA3.1(+) mammalian expression plasmid (Invitrogen). It was named pCMV-*NQO1*. The CMV promoter in pCD-NQO1 was replaced by the 2.4 kb wild-type or SNP-human *NQO1* promoter, which was excised from pGL4-*NQO1* and pGL4-_SNP_*NQO1*. The two new plasmids were named p*NQO1-NQO1* and p_SNP_*NQO1* (or pSNP). The correct sequence of each plasmid was verified by DNA sequencing.

### 2.3. Stable Expression of NQO1 in BEAS-2B Cells

pcDNA3.1, pCMV-*NQO1*, p*NQO1-NQO1*, or pSNP was transfected into BEAS-2B cells using SuperFect (Qiagen) and maintained in 100 *μ*g/ml Geneticin (Invitrogen). Clones were screened by immunofluorescence staining with the A180 NQO1 antibody (Santa Cruz Biotechnology) and verified by qPCR. These 4 stable transfected BEAS-2B cell lines were named Ctr-, CMV-*NQO1*-, *NQO1-NQO1*-, and SNP-BEAS-2B cells, respectively.

### 2.4. NQO1 Assay

This method was adapted from Tsvetkov et al. in 2005 [30]. Cells were lysed in 25 mM Tris, pH 7.5/1 mM EDTA/0.1 mM dithiothreitol (DTT). Cell lysate (30-50 *μ*g) was mixed in 200 *μ*l of reaction buffer (25 mM Tris-HCl (pH 7.5), 0.01% Tween 20, 0.7 mg/ml BSA (pH 7.4), 40 *μ*M menadione, 5 *μ*M flavin adenine dinucleotide (FAD), and 200 *μ*M nicotinamide adenine dinucleotide (NADH)) in a 96-well plate. Absorbance at 340 nm (*A*_340nm_) was measured repeatedly during the decay of NADH. Statistical difference between each group was calculated with Tukey's multiple comparison test in repeated measures ANOVA using GraphPad Prism 5.

### 2.5. qPCR

Total RNA was extracted from the cell lysates using the Qiagen RNeasy Kit. The mRNA level was quantified with the BioRad iScript Reverse Transcription Supermix and the iQ SYBR Green Supermix RT-qPCR method, while the primers for *CYP1B1* and the reference gene *OAZ1*were obtained following the method of Dinu et al. in 2016 [31]. Primers for *AHR*, *CYP1A1*, and *NQO1* were obtained following the method of Shivanna et al. in 2011 [32]. Other primers included the following: *NME1*, tcattgcgatcaaaccagat and caacgtagtgttccttgaga; *PCNA*, aggcactcaaggacctcatca and gagtccatgctctgcaggttt; *ERCC1*, ggcgacgtaattcccgacta and agttcttccccaggctctgc; *OGG1*, gatgttgttgttggaggaa and aagaggtggctcagaaat; *XPC*, taaatagcaaatctcctttcc and acacctactacctctcaa; *PARP1*, cacttgctgcttgttgaa and gaacgacctgatctggaa; *DDB2*, gcattctgagattccaaagc and tgtagcctggatgtgtct; *XAB2*, cccccaaaatatgccaagacct and tgctcgtccgacagcacctc; and *NEIL2*, gcactcaggactgaaccga and ctgtctgctatacactgctgga.

### 2.6. Cell Viability Assays

Cell viability was determined by the MTT (3-(4,5-dimethylthiazol-2-yl)-2,5-diphenyltetrazolium bromide) Proliferation Assay Kit from ATCC and the live protease assay using the ApoTox-Glo Triplex Assay Kit from Promega, according to the manufacturers' instructions and the method of Dinu et al. in 2016 [31].

### 2.7. ApoTox-Glo Triplex Assay

Cytotoxicity and cell viability of cells in 96-well black-walled plates were determined using the ApoTox-Glo Triplex Assay (Promega) according to the manufacturers' instructions and the method of Dinu et al. in 2016 [31]. Cell viability (live cell protease activity) and dead cell level (dead cell protease activity) were determined by fluorescence absorption at 505 nm and 520 nm, respectively. Caspase 3/7 assays were determined by bioluminescence as reported earlier [31].

### 2.8. Knockdown of CYP1A1 in Ctr and NQO1-NQO1 Cells

Ctr and *NQO1-NQO1* cells were transfected with human CYP1A1 siRNA (Thermo Fisher Scientific #4392420, Assay ID s3800) or negative control siRNA (Thermo Fisher Scientific #4390843) using the Lipofectamine RNAiMAX Transfection Reagent (Invitrogen) according to the manufacturers' instructions. The cells were subjected to hyperoxia treatment 24 h after the transfection.

### 2.9. Detection of Oxidative DNA Lesions by the ^32^P-Postlabeling Assay

BEAS-2B human cells were grown in culture and transfected with pcDNA3.1, pCMV-*NQO1*, pNQO1-*NQO1*, or pSNP. Cells were exposed to 80% oxygen or room air for 48 hours. DNA was extracted from the cells and subjected to enzymatic digestion and enrichment of the oxidative products (pNp-cAP) from the DNA digest. Dinucleotide adducts were labeled with [^32^P]-orthophosphate from [*γ*-^32^]-ATP mediated by polynucleotide kinase and then separated by two-dimensional thin-layer chromatography per the previously described method [16, 33–36]. The labeled nucleotides were chromatographed on polyethyleneimine-impregnated cellulose thin-layer chromatography (TLC) plates and imaged by the InstantImager (Packard Instruments, Merien, Connecticut). Levels of total 8,5-*cyclo*-20-deoxyadenosine (cA) oxidative DNA adducts as well as the individual dinucleotides adenine cA (AcA), guanine cA (GcA), cytosine (GcA), and thymine cA (TcA) were analyzed as reported previously [25, 35]. ^32^P-labeled DNA adducts were quantified by InstantImager [35, 36]. The oxidative dinucleotide adducts of cells on TLC maps were identified by comparing with those from genomic DNA obtained from endotracheal aspirate of an ARDS patient who was subjected to supplemental oxygen and mechanical ventilation. The ARDS patient sample was obtained from Ben Taub General Hospital, Houston, TX, as part of an ongoing IRB-approved study at Baylor College of Medicine.

### 2.10. Detection of 8-Hydroxy-2′-Deoxyguanosine (8-OHdG) by LC-MS/MS

Total DNA was isolated from cells using proteinase K digestion followed by phenol/chloroform extraction and ethanol precipitation. After undergoing a series of digestion with micrococcal endonuclease, spleen phosphodiesterase, nuclease P1, and calf intestinal phosphatase, 0.2 *μ*g DNA in 50 *μ*l of a 1 : 1 methanol/water mixture was subjected to LC-MS/MS analysis [37, 38].

### 2.11. Statistical Analyses

All data were analyzed by comparing mean ± SE of at least 3 independent experiments. Mean values among different groups were compared using Student's *t*-test, unless specified, and *P* < 0.05 was considered significant.

## 3. Results

### 3.1. Effect of Hyperoxia on NQO1, CYP1A1, CYP1B1, and AHR Gene Expression in Control and NQO1 Overexpressing Cells

**Figure 1:**
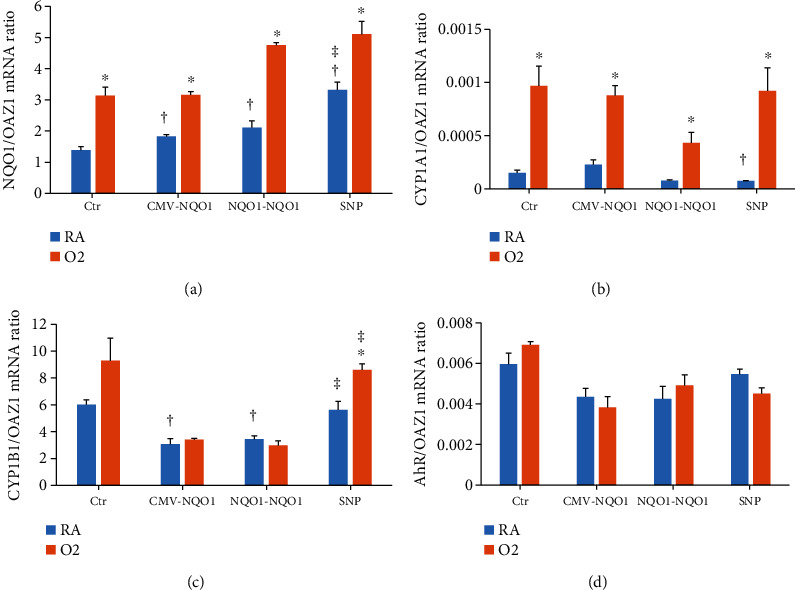
Overexpression of *NQO1* in *NQO1*-stable transfected cells. BEAS-2B cells stably transfected with pcDNA3.1 (Ctr), pCD-*NQO1* (CMV-*NQO1*), p_WT-NQO_*NQO1* (*NQO1-NQO1*), and p_mut-NQO_*NQO1* (SNP) were incubated under room air (RA) or 80% O_2_ (O_2_) conditions for 48 h and subjected to qPCR using total RNA extracted from these cells. Gene expression of *NQO1* (a), *CYP1A1* (b), *CYP1B1* (c), and AHR (d) were determined. ^∗^Statistically significant difference between room air and hyperoxia. ^†^Statistically significant difference with Ctr. ^‡^Statistically significant difference between the *NQO1-NQO1* and SNP-*NQO1* promoter (*n* = 3; *P* < 0.05).

Stable cell lines transfected with pcDNA3.1 (Ctr), pCMV-*NQO1* (CMV-*NQO1*), p_NQO1_*NQO1* (*NQO1-NQO1*), and p_SNP_*NQO1* (SNP) were cultured under room air (RA) or 80% oxygen (O_2_) for 48 h. qPCR, using *OAZ1* as the reference gene, indicated that *NQO1* mRNA level was significantly induced by hyperoxia in Ctr cells (Figure 1(a)). In cells stably transfected with *NQO1*-containing cDNA plasmids, hyperoxia augmented the NQO1 expression by 77%, 118%, and 66% in CMV-*NQO1*, *NQO1-NQO1*, and SNP cells, respectively, compared to room air conditions (Figure 1(a)). NQO1 mRNA level was significantly higher in each of the NQO1 overexpressed cells compared to Ctr cells even in room air (Figure 1(a)), with SNP cells showing greater NQO1 expression compared to *NQO1-NQO1* cells. The extent of NQO1 induction from baseline levels in SNP cells by hyperoxia appeared to be less than that of *NQO1-NQO1* cells (Figure 1(a)).

**Figure 2:**
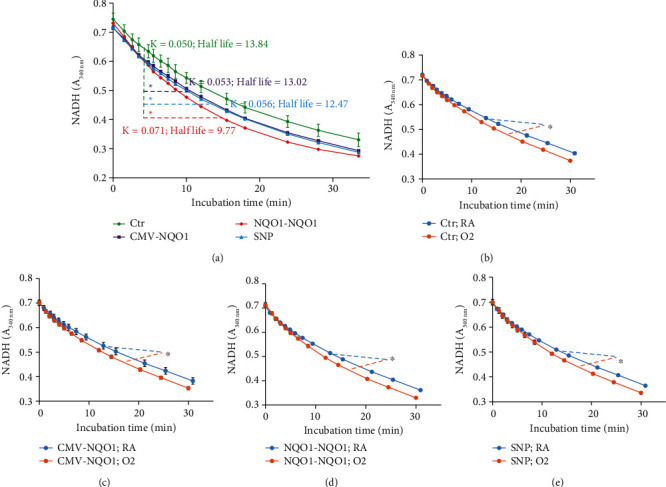
NADH decay curve indicated enhanced NQO1 enzyme activity in cells stably transfected with NQO1 cDNA (a), or by hyperoxia (b–e). (a) 50 *μ*g lysate from each of the stably transfected BEAS-2B cell lines Ctr, *NQO1-NQO1*, and SNP was subjected to the NQO1 assay. (b–e) Four cell lines were incubated under room air (RA) or 80% O_2_ (O_2_) conditions for 48 h. 30 *μ*g lysate was subjected to the NQO1 assay. One way ANOVA indicated statistically significant difference between specified curves. *K*_decay_ value and half-life were the curve fitting results using the “one phase decay” model in GraphPad Prism 5. ^∗^Statistically significant difference with Ctr cells (a) or between RA and O_2_ (b–e) (*n* = 3; *P* < 0.05).

At protein level, NQO1 overexpression was detected by the NQO1 assay (Figure 2(a)). NADH decay was measured by *A*_340nm_ in 50 *μ*g lysate protein from Ctr cells as well as CMV-*NQO1*, *NQO1-NQO1*, and SNP cells in the presence of menadione (substrate) and FAD (coenzyme), a reaction catalyzed by the NQO1 enzyme in the lysate. The decay of NADH appeared to be significantly faster in CMV-*NQO1*, *NQO1-NQO1*, and SNP cells when comparing with Ctr cells, which was represented by slightly but statistically significant higher *K*_decay_ value and shorter half-life (Figure 2(a)). This result indicated that CMV-*NQO1*, *NQO1-NQO1*, and SNP cells expressed higher NQO1 activities than Ctr cells.

The NQO1 assay also showed that hyperoxia (80% O_2_, 48 h) significantly induced the NQO1 enzyme in CMV-*NQO1*, *NQO1-NQO1*, and SNP cells when comparing with Ctr cells (Figures 2(b)–2(e)). Western blot analyses detected NQO1 protein in each of the constructs (Supplemental Figure 1), and hyperoxia induced NQO1 expression in each of the cells, albeit the basal expression of NQO1 was not different among the constructs.

At mRNA level, hyperoxia elicited marked induction of *CYP1A1* (~10-fold) in Ctr cells and CMV-NQO1 cells (~5-fold) (Figure 1(b)). Hyperoxia also caused induction of *CYP1A1* in *NQO1-NQO1* and SNP cells (Figure 1(b)), with the extent of induction being greater in the SNP cells (~20-fold). Hyperoxia induced *CYP1B1* gene expression in SNP cells but not in the other cell lines (Figure 1(c)). *CYP1B1* expression in room air in the CMV-*NQO1* and *NQO1-NQO1* was lower than Ctr cells. On the other hand, CYP1B1 expression in SNP cells was higher than that of *NQO1-NQO1* in both room air and hyperoxic conditions (Figure 1(c)). The expression of AHR gene was not altered by hyperoxia in any of the cells (Figure 1(d)).

### 3.2. Overexpression of NQO1 Altered Hyperoxic Cytotoxicity

**Figure 3:**
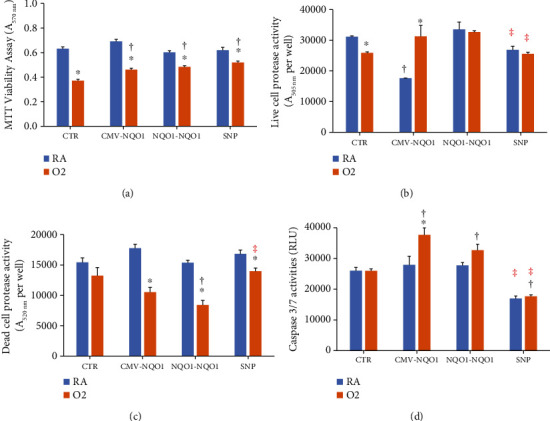
*NQO1* overexpression protected cells from hyperoxic toxicity. BEAS-2B cells stably transfected with pcDNA3.1 (Ctr), pCMV-*NQO1* (CMV-*NQO1*), p_WT-NQO_*NQO1* (*NQO1-NQO1*), and p_SNP-NQO_*NQO1* (SNP) were incubated under room air (RA) or 80% O_2_ (O_2_) condition for 48 h and the MTT cell viability assay (a), the live cell protease activity assay (b), the dead cell protease activity assay (c), and the caspase 3/7 activity assay (d) were determined using the Promega ApoTox-Glo Triplex Assay. ^∗^Statistically significant differences between the RA and O_2_ groups. ^†^Statistically significant difference with Ctr. ^‡^Statistically significant difference between the WT- and SNP-*NQO1* promoter (*n* = 3; *P* < 0.05). Hyperoxia decreased cell viability, which was attenuated by overexpression of NQO1 (a and b).

Hyperoxia significantly decreased cell viability. The *A*_590nm_ of the Ctr cells decreased by 41% in the MTT assay (Figure 3(a)). Overexpression of NQO1 resulted in improvement in cell viability (16-33%) in the 3 NQO1-overexpressed cell lines. The live cell protease assay (Figure 3(b)) exhibited a comparable result, in which hyperoxia decreased cell viability, and it was rescued in part by overexpression of NQO1, with CMV-*NQO1* cells showing an increase in cell viability after hyperoxia. The dead cell protease activities represented the number of dead cells in the wells. In *NQO1-NQO1* (and CMV-*NQO1*) cells, cell death was 45-50% lower compared to room air controls, and as in *NQO1-NQO1* cells, cell death in hyperoxic cells was lower than that in the Ctr group (Figure 3(c)). Cell death was also decreased in SNP cells by hyperoxia, but the number of dead cells were higher in SNP cells exposed to hyperoxia compared to those of *NQO1-NQO1* (Figure 3(c)). Interestingly, there was an increase of caspase 3/7 activities in the live cells (Figure 3(d)) overexpressing NQO1. This result suggested that overexpression of NQO1 might redirect the hyperoxia-stressed cells into an apoptotic pathway rather than necrotic death. This redirection was decreased in cells harboring the A-1221C SNP on the *NQO1* promoter because SNP cells appeared to not be different from Ctr cells (Figure 3(d)). In all these experiments, an equal number of cells were plated from all cell lines.

### 3.3. Effect of Hyperoxia on Oxidative DNA Adduct Formation

**Figure 4:**
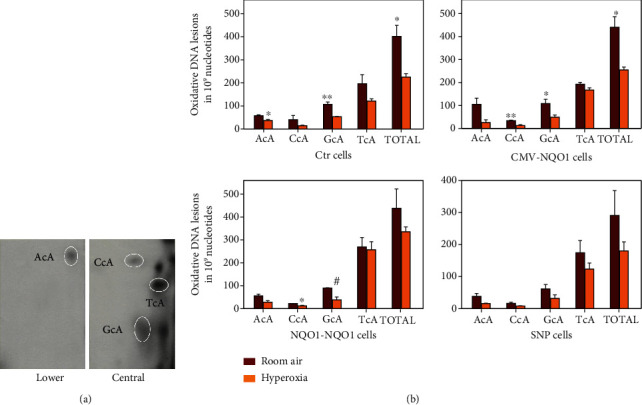
Hyperoxia decreased DNA adduct formation in ^32^P-postlabeling experiments, and high cellular levels of NQO1 and CYP1A1 might attenuate such effect. (a) A typical result of a ^32^P-postlabeling experiment showing chromatographic images labeled with AcA, GcA, CcA, and TcA. (b) Quantitation of the ^32^P-postlabeling experiment. Three stably transfected BEAS-2B cell lines Ctr, CMV-*NQO1*, *NQO1-NQO1*, and SNP (see Figure 2) were incubated under RA or O_2_ for 48 h, followed by DNA extraction and ^32^P-postlabeling experiments (*n* = 3; ^∗^*P* < 0.05; ^∗∗^*P* < 0.01; ^#^*P* = 0.05).

Previous studies have shown that hyperoxia increases oxidative DNA adduct formation [34]. Levels of total 8,5-*cyclo*-2-deoxyadenosine (cA) oxidative DNA adducts as well as the individual dinucleotides adenine cA (AcA) and guanine cA (GcA) were determined by Veith et al. in 2018 [34] and by Zhou and Moorthy in 2015 [35] (Figure 3(a)). The DNA in Figure 4(a) was obtained from an endotracheal aspirate sample from an ARDS patient as described under Materials and Methods. The cA adducts are formed by hydroxyl radical attack on 2′-deoxyadenosine, which then binds covalently with the adjacent nucleotide [33, 35]. The location of these adducts on the thin-layer chromatography (TLC) plates was based on cochromatography and rechromatography using structurally characterized adducts [36]. Total pulmonary adducts were quantified in Figure 4(b), which included the aggregate values of the AcA, CcA, GcA, and TcA adducts. The individual dinucleotide adducts were also analyzed as well. Our main finding was that in all cells, the formation of the DNA adducts AcA, CcA, GcA, and TcA was mostly decreased in the hyperoxia groups. The hyperoxia-mediated decrease in total adduct levels was significant in Ctr cells and CMV-*NQO1* cells but not significant in *NQO1-NQO1* or SNP cells (Figure 4(b)).

### 3.4. Inverse Correlation between Oxidative DNA Adducts and CYP1A1/NQO1 Expression

**Figure 5:**
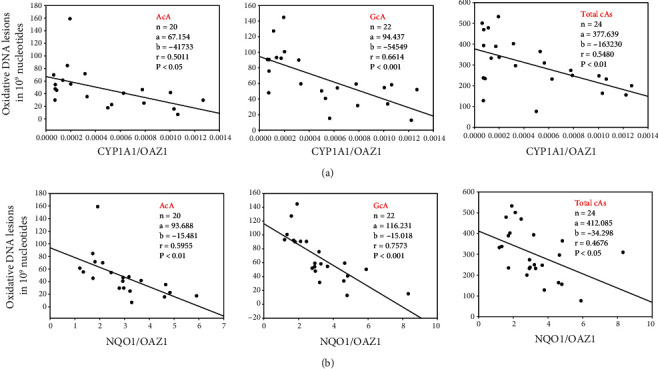
Linear correlations between levels of 8,5′-cyclopurine-2′-deoxynucleotides (oxidative DNA lesions in 10^9^ normal nucleotides) and CYP1A1/OAZ1 (a) or NQO1/OAZ1 (b) in lung cell lines. Data of DNA adducts from all the individual samples (*n* = 20‐24) in room air or hyperoxic condition in each cell line were combined and plotted against the mean *CYP1A1* (a) or *NQO1* (b) gene expression using data from all individual samples. Significant inverse correlations were observed between levels of AcA, GcA, and Total cA (sum of AcA, CcA, GcA, and TcA) and *CYP1A1*/OAZ1 (a) or *NQO1*/OAZ1 (b).

In order to determine if a mechanistic relationship exists between oxidative DNA adducts and gene expression of *CYP1A1* or *NQO1*, we conducted a regression analyses between levels of each of the cyclopurines (AcA, GcA, TcA, and CcA) and total adducts, and that of *CYP1A1* (Figure 5(a)) or *NQO1* (Figure 5(b)). The data were compared among these parameters after combining the data obtained from at least 3 individual experiments in all the 4 cell lines, which were either maintained in room air or exposed to hyperoxia (Figure 5). The results showed that each of the cyclopurine dinucleotides and total adducts inversely correlated with *CYP1A* (Figure 5(a)) or *NQO1* (Figure 5(b)) gene expression.

### 3.5. Effect of Hyperoxia on 8-OHdG Levels

**Figure 6:**
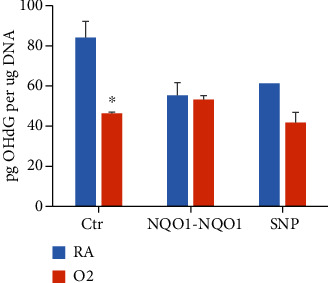
Hyperoxia or NQO1 overexpression decreased 8-OHdG formation. Three stably transfected BEAS-2B cell lines Ctr, *NQO1-NQO1*, and SNP were incubated under RA or O_2_ for 48 h. Genomic DNA was isolated from the cells and digested with micrococcal endonuclease, spleen phosphodiesterase, nuclease P1, and calf intestinal phosphatase, followed with LC-MS of 8-OHdG using 0.2 *μ*g enzyme-treated DNA (*n* = 3; ^∗^*P* < 0.05).

In order to determine if the oxidative DNA adduct data correlated with 8-OHdG levels, we measured 8-OHdG levels under similar experimental conditions by LC-MS/MS in the total DNA extracted from Ctr, *NQO1-NQO1*, and SNP cells. The 8-OHdG level in the genomic DNA from Ctr cells was significantly (34.2%) decreased by hyperoxia, and this decrease was not observed in *NQO1-NQO1* cells or the SNP cells (Figure 6).

### 3.6. Role of CYP1A1 in the Modulation of Cell Toxicity and 8-OHdG

**Figure 7:**
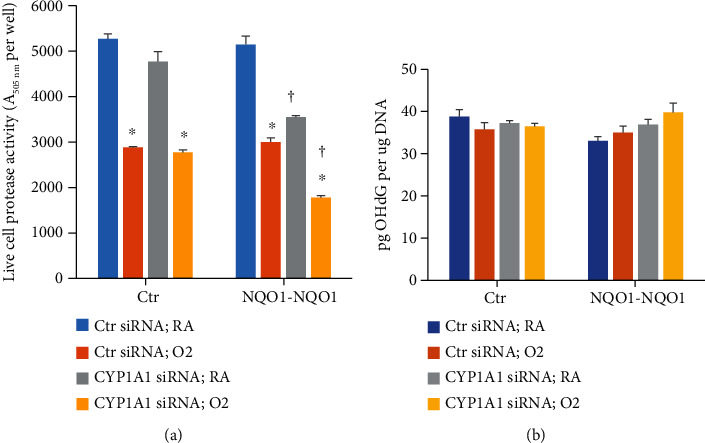
Effect of *CYP1A1* silencing on cell viability (a) and 8-OHdG (b) levels. Stably transfected BEAS-2B cell lines Ctr or *NQO1-NQO1* were transfected with *CYP1A1* siRNA or control siRNA and incubated under RA or O_2_ for 48 h, followed by determination of live cell protease activity using the Promega ApoTox-Glo Triplex Assay (a) or the LS-MS/MS assay (b) (*n* = 3; ^∗^*P* < 0.05 by Student's *t*-test.

In order to determine if inhibiting *CYP1A1* would modulate the cell toxicity and oxidative DNA damage responses by hyperoxia, we determined cell viability (Figure 7(a)) and levels of 8-OHdG (Figure 7(b)) in Ctr and *NQO1-NQO1* cells that had been treated with control or *CYP1A1* siRNA. *CYP1A1* mRNA knockdown by *CYP1A1* siRNA was verified by qPCR (Supplemental Figure 2). As shown in Figure 7(a), hyperoxia decreased cell viability in cells treated with control or *CYP1A1* siRNA. In *NQO1-NQO1* cells, the decrease in cell viability by hyperoxia was higher in those that were treated with *CYP1A1* siRNA (Figure 7(a)) compared to those treated with control siRNA, suggesting that *CYP1A1* might protect the cell from hyperoxia in an *NQO1*-dependent manner.

In order to study the role of *CYP1A1* on oxidative DNA damage, we measured 8-OHdG levels in Ctr and NQO1 cells that were treated with control or *CYP1A1* siRNA. As shown in Figure 7(b), there were no significant changes in 8-OHdG levels in Ctr cells that were treated with *CYP1A1* siRNA. However, in *NQO1-NQO1* cells, 8-OHdG was slightly increased by hyperoxia by about 7% and by 12% in cells that were treated with *CYP1A1* siRNA compared to those that were treated with control siRNA (Figure 7(b)), although the results were not statistically significant.

### 3.7. Modulation of DNA Repair Genes by Hyperoxia

**Figure 8:**
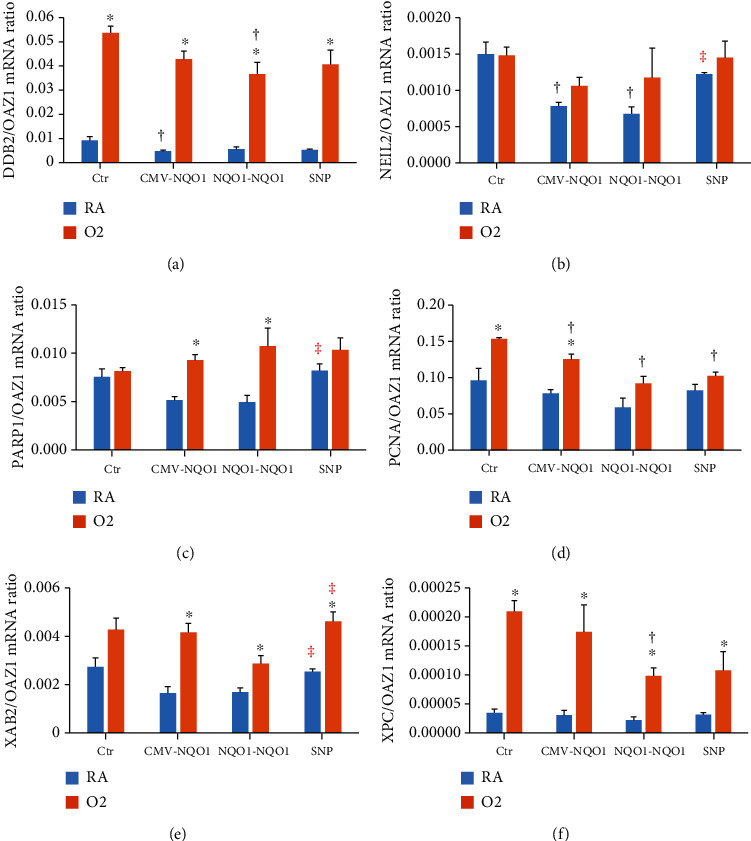
Effect of hyperoxia on DNA repair genes. Four stably transfected BEAS-2B cell lines Ctr, CMV-*NQO1*, *NQO1-NQO1*, and SNP were incubated in RA or O_2_ for 48 h and subjected to qPCR. ^∗^Statistically significant difference between RA and O_2_ groups. ^†^Statistically significant difference compared to Ctr. ^‡^Statistically significant difference between *NQO1-NQO1* and SNP (*n* = 3; *P* < 0.05).

We determined the effect of hyperoxia on a number of DNA repair genes to test the hypothesis that hyperoxia would induce DNA repair enzymes, which could play a protective role against oxygen toxicity. We also wanted to determine if DNA repair genes could in part explain the decrease in oxidative DNA damage caused by hyperoxia and also if NQO1 overexpression would modulate DNA repair mechanisms. Exposure to hyperoxic conditions significantly increased the expression of damage-specific DNA binding protein 2 (*DDB2)* and xeroderma pigmentosum, complementation group C (*XPC*) (Figures 8(a) and 8(f)) in Ctr, CMV-*NQO1*, *NQO1-NQO1*, and SNP-transfected cells. In hyperoxic cells, significantly less *XPC* expression was detected in cells transfected with *NQO1-NQO1* promoter constructs compared to control cells, whereas hyperoxic cells transfected with SNP-containing promoter constructs had levels of *XPC* expression that were not significantly different from control-transfected cells (Figure 8(f)). Nei-like DNA glycosylase 2 (*NEIL2*) expression (Figure 8(b)) did not show any increase with hyperoxia; however, the expression was decreased under normoxic conditions in CMV-*NQO1* and *NQO1-NQO1* cells compared to Ctr cells.

Poly(ADP-ribose polymerase 1 (*PARP1*) expression was increased in CMV-NQO1 and NQO1-NQO1 cells upon exposure to hyperoxia (Figure 8(c)). In cells with SNP, PARP1 expression was higher than NQO1-NQO1 cells under normoxia (Figure 8(c)). Hyperoxia caused induction of proliferating cell nuclear antigen (*PCNA*) under hyperoxic conditions in Ctr and CMV-NQO1 but not in NQO1-NQO1 or SNP-containing cells (Figure 8(d)). Under hyperoxia, the expression of *PCNA* was lower in CMV-NQO1, NQO1-NQO1, and SNP-containing cells compared to Ctr cells. Additionally, there were significantly increased levels of XPA binding protein 2 (*XAB2*) mRNA levels in hyperoxia-exposed CMV-*NQO1*, *NQO1-NQO1*, and SNP cells with levels being relatively higher in the latter compared to *NQO1-NQO1* cells (Figure 8(e)). In cells carrying the SNP, the expression was increased in normoxia as well as compared to *NQO1-NQO1* cells (Figure 8(e)).

## 4. Discussion

The overall goal of this study was to determine the role of human NQO1 in hyperoxia-mediated cellular injury and oxidative DNA damage. Specifically, we tested the hypothesis that overexpression of NQO1 in BEAS-2B cells will mitigate cell injury and oxidative DNA damage caused by hyperoxia and that A-1221C SNP in the *NQO1* promoter would display altered susceptibility to hyperoxia-mediated toxicity.

Our results showing increased hyperoxia-mediated NQO1 expression in Ctr cells and in cells overexpressing NQO1 in CMV-*NQO1* and *NQO1-NQO1* cells (Figure 1(a)) were in agreement with earlier studies showing induction of NQO1 by hyperoxia [29, 39]. Our observation that SNP cells showed lesser extent induction of NQO1 expression by hyperoxia compared to *NQO1-NQO1* cells was probably due to the regulatory elements in the SNP construct that were masked, leading to reduced induction of the gene (Figure 1(a)). However, we did see increased NQO1 expression per se in the SNP cells exposed to hyperoxia, and additional work needs to be done to explain this discrepancy. The induction of the *CYP1A1* gene by hyperoxia (Figure 1(b)) was in agreement with earlier reports of induction of the CYP1A1 enzyme *in vitro* [40] and *in vivo* [13–17]. The suppression of induction of *CYP1A1* in *NQO1-NQO1* cells was probably due to the metabolism of ROS-mediated AHR ligands [41] that contributed to CYP1A1 enhancement by hyperoxia [34]. The restoration of *CYP1A1* induction in the SNP cells by hyperoxia (Figure 1(b)) could have been due to an increase in ROS levels in these cells, which in turn may have resulted in increased formation of endogenous ligands that contributed to *CYP1A1* induction by hyperoxia.

The suppression of *CYP1B1* gene expression (Figure 1(c)) in CMV-*NQO1* and *NQO1-NQO1* cells in room air conditions could be explained by the metabolism of ROS-mediated endogenous AHR ligands that were responsible for CYP1B1 induction probably by CYP1A1. The fact that CYP1B1 expression was restored in SNP cells in room air and was induced in these cells by hyperoxia lends credence to the theory that endogenous AHR ligands contributed to CYP1B1 induction.

The fact that the decay of NADH was significantly faster in CMV-*NQO1*, *NQO1-NQO1*, and SNP cells compared to Ctr cells (Figure 2(a)) suggested that CMV-*NQO1*, *NQO1-NQO1*, and SNP cells expressed higher NQO1 activities than Ctr cells. Given that NQO1 is an antioxidant enzyme, we first sought to evaluate the role of oxygen toxicity in human lung cells that had been transfected with the WT- (*NQO1-NQO1*) and SNP-containing *NQO1* promoter/gene construct compared to controls. Cells that had not been transfected with the *NQO1* constructs displayed decreased cell viability, decreased live cell protease, and increased cell death under hyperoxic conditions (Figures 3(a)–3(c)), suggesting that oxidative stress contributed to cell injury. In the live cell and dead cell protease assays (Figures 3(b) and 3(c)), cells transfected with the constitutively active CMV promotor/*NQO1*gene construct demonstrated enhanced ratio of live/dead cell protease activities under hyperoxic conditions compared to room air, which implied that the overexpression of CMV-NQO1 might prevent the disruption of the cell membrane and keep the proteases inside the cells. In cells transfected with SNP A-1221C, the live cell protease activity was lesser in both room air and hyperoxic conditions compared to the *NQO1-NQO1* group (Figure 3(b)), probably due to a partial loss of protection to cell membrane integrity by NQO1 due to the SNP.

On the other hand, both CMV and *NQO1-NQO1* cells showed significantly decreased dead cell protease activities under hyperoxic conditions, which was probably due to protection of cell membrane integrity by NQO1 overexpression in these cells (Figure 3(c)). Figure 3(d) shows the increase of caspase 3/7 activities by hyperoxia in CMV-*NQO1* and *NQO1*-*NQO1* cells. This increase suggested that part of the hyperoxia-damaged cells might have entered an apoptotic pathway. This would also explain why the CMV and *NQO1-NQO1* cells exhibited increased live cell protease activities compared to Ctr cells under hyperoxic conditions (Figure 3(b)).

To further characterize the toxic effect of high levels of oxygen exposure on cells transfected with the various *NQO1* promoter/gene constructs, we investigated the effect of hyperoxia on oxidative DNA lesions by ^32^P-postlabeling. Our observations (Figure 4(b)) showing decreased levels of AcA, GcA, and total adducts in all cells were surprising, as we would expect increased oxidative DNA damage by hyperoxia due to increased oxidative stress.

Firstly, we found an inverse correlation between oxidative DNA adducts and CYP1A1 and NQO1 gene expression (Figures 5(a) and 5(b)). This observation supports the hypothesis that these enzymes are protective against oxidative DNA damage. Our numerous studies in animal models [13–19, 42] have clearly shown the role of both CYP1A1 and NQO1 in the protection against oxidative injury. Our recent study [19] showing the increased susceptibility to hyperoxic lung injury of mice lacking the gene for *nrf2*, and the rescue of this phenotype by the CYP1A1 inducer *β*-napthoflavone, lends further credence to the hypothesis that both Nrf2-regulated enzymes (e.g., NQ01) and CYP1A enzymes play a beneficial role in oxygen injury. While CYP1A1 might protect the cells from oxidative stress by metabolizing toxic lipid hydroperoxides [16–20], it is possible that NQO1 in the current study might have protected cells from oxidative stress by metabolizing quinones and semiquniones [21, 22]. The innovative aspect of our current study is that our results show a decrease in the extent of induction of CYP1A1 by hyperoxia in NQO1-NQO1 cells, suggesting a role for NQO1 in the regulation of CYP1A1 expression.

Our results showing the attenuation of 8-OHdG by hyperoxia (Figure 6) in Ctr cells but not in *NQO1-NQO1* or SNP cells were in agreement with our studies on bulky oxidative lesions (Figure 4). Although studies reported in the literature show increased levels of OHdG in rat alveolar type II cells exposed to hyperoxia [43], Jin et al. [44] showed that human 8-oxoguanine DNA glycolyase increases resistance to hyperoxic toxicity in alveolar epithelial A549 cells. In our studies, it is possible that hyperoxia in BEAS-2B cells caused a decrease in OHdG levels in part by inducing DNA repair.

Because hyperoxia-mediated induction of DNA repair pathways [45] could in part play a role in the attenuation of oxidative DNA lesions by hyperoxia in Ctr cells (Figures 4 and 6), we determined the effect of hyperoxia on base excision repair (BER) as well as nucleotide excision repair pathways. We studied NEIL2, PARP1, and PCNA as representative of the BER pathway and DDB2, XAB2, and XPC as representative of the NER pathway [46]. While 8-OHdG is repaired by BER [44], the oxidative DNA adducts are repaired by NER mechanisms [36, 47]. Our observations showing a marked induction of DDB2 and XPC by hyperoxia in Ctr cells (Figure 8) supported the idea that hyperoxia in part caused attenuation of bulky oxidative DNA lesions by enhancing NER pathways. The decrease of 8-OHdG by hyperoxia in Ctr cells (Figure 6), but not in *NQO1-NQO1* cells or SNP cells, was probably due to significant induction of the proliferating cell nuclear antigen (PCNA), which repairs DNA via BER in Ctr but not in *NQO1-NQO1* or SNP cells (Figure 8(d)). Also, the induction of XPC, a NER enzyme, was induced by hyperoxia to a much higher degree in Ctr than *NQO1-NQO1* or SNP cells (Figure 8(f)). Thus, we observed a significant modulation of both BER and NER genes by hyperoxia in CMV-*NQO1*, *NQO1-NQO1*, and SNP cells.

We did not see a striking difference of DNA repair gene expression among the *NQO1-NQO1* and SNP cells, suggesting that the SNP A-1221C did not play a major role in the regulation of DNA repair pathways. Our finding that the protection against hyperoxic toxicity in SNP cells was partially lost in spite of these cells having high NQO1 mRNA (Figure 1(a)) could have been due to the fact that this SNP produced a gene product that had lower NQO1 activity. Previous reports have implicated *NQO1* promotor SNPs, specifically the A-1221C SNP, as having a potential protective effect on the severity of acute lung injury in patients suffering from ALI/ARDS [29]. That we did not observe a similar protective effect could have been due to the fact that the current study was in the human BEAS-2B cell line that was exposed to hyperoxia (80% O_2_ and 5% CO_2_) for 48 h, and that mechanisms independent of NQO1 may have contributed to the protective effects in humans expressing the SNP A 1221C variant. Future successful creation of *in vivo* knock-in mouse models that carry the wild-type NQO1 or the A-1221C SNP will help us delineate the mechanistic role of A-1221C SNP in oxygen toxicity in relation to ARDS.

In summary, our data support a protective role for human NQO1 against oxygen-mediated toxicity and oxidative DNA lesions in human pulmonary cells, and this protection is partially lost in cells carrying the A-1221C SNP. Moreover, we also demonstrate a novel protective role for CYP1A1 in the attenuation of oxidative cell and DNA injury. Future studies on the mechanisms of attenuation of oxidative injury by NQO1 should help in developing novel approaches for the prevention/treatment of ARDS in humans.

## Data Availability

The data used to support the findings of this study are available from the corresponding author upon request.

## Supplementary Materials

Supplementary Materials Supplementary Materials S1 Hyperoxia increased NQO1 protein expression. BEAS-2B cells stably transfected with pcDNA3.1 (Ctr), pCD-*NQO1* (CMV-*NQO1*), pWT-*NQO1-NQO1* (NQO1-NQO1), and *pmut-NQO1-NQO1* (SNP) were incubated under room air (RA) or 80% O_2_ conditions for 48 h and subjected to western blotting using 20 *μ*g total protein of cell lysates per well and 1 : 1000 dilution of A-180 NQO1 antibody (Santa Cruz Biotechnology). (A) Colorimetry image of a representative blot. (B) Densitometry analysis of 2 replicate blots. The result indicated that hyperoxia slightly induced NQO1 protein expression in all 4 cell lines. However, it did not show increased basal NQO1 expression in any of the three NQO1-overexpressed cell lines. S2. *CYP1A1* siRNA downregulated CYP1A1 mRNA in both Ctr cells and *NQO1-NQO1* cells. Cells were transfected with control siRNA or *CYP1A1* siRNA and cultured in RA condition for 48 h. Total RNA were extracted and subjected to qPCR of *CYP1A1* and the reference gene *OAZ1*. The Ct value of *CYP1A1* was not detected in one group of sample, probably “Ct value > 39.” (*n* = 1‐3).
