# Supplementary material for: Role of Human NADPH Quinone Oxidoreductase (NQO1) in Oxygen-Mediated Cellular Injury and Oxidative DNA Damage in Human Pulmonary Cells
Source: Oxid Med Cell Longev. 2021 Oct 15;2021:5544600. doi: 10.1155/2021/5544600 (PMC8536466; doi:10.1155/2021/5544600)
Supplement: Supplementary Materials — S1 Hyperoxia increased NQO1 protein expression. BEAS-2B cells stably transfected with pcDNA3.1 (Ctr), pCD-NQO1 (CMV-NQO1), pWT-NQO1-NQO1 (NQO1-NQO1), and pmut-NQO1-NQO1 (SNP) were incubated under room air (RA) or 80% O2 conditions for 48 h and subjected to western blotting using 20 μg total protein of cell lysates per well and 1 : 1000 dilution of A-180 NQO1 antibody (Santa Cruz Biotechnology). (A) Colorimetry image of a representative blot. (B) Densitometry analysis of 2 replicate blots. The result indicated that hyperoxia slightly induced NQO1 protein expression in all 4 cell lines. However, it did not show increased basal NQO1 expression in any of the three NQO1-overexpressed cell lines. S2. CYP1A1 siRNA downregulated CYP1A1 mRNA in both Ctr cells and NQO1-NQO1 cells. Cells were transfected with control siRNA or CYP1A1 siRNA and cultured in RA condition for 48 h. Total RNA were extracted and subjected to qPCR of CYP1A1 and the reference gene OAZ1. The Ct value of CYP1A1 was not detected in one group of sample, probably “Ct value > 39.” (n = 1‐3). [file 5544600.f1.zip › 210908_Oxidative_Medicine_Suppl_Figures.docx]

Supplemental Figure 1

Supplemental Figure 2

S1. Hyperoxia increased NQO1 protein expression. BEAS-2B cells stably transfected with pcDNA3.1 (Ctr), pCD-*NQO1* (CMV-*NQO1*), pWT-*NQO1-NQO1* (NQO1-NQO1), and *pmut-NQO1-NQO1* (SNP) were incubated under room air (RA) or 80% O_2_ conditions for 48 h and subjected to western blotting using 20 µg total protein of cell lysates per well and 1:1000 dilution of A-180 NQO1 antibody (Santa Cruz Biotech). (A) Colorimetry image of a representative blot; (B) Densitometry analysis of 2 replicate blots. The result indicated that hyperoxia slightly induced NQO1 protein expression in all 4 cell lines. However, it didn’t show increased basal NQO1 expression in any of the three NQO1-overexpressed cell lines.

S2. *CYP1A1* siRNA down-regulated CYP1A1 mRNA in both Ctr cells and *NQO1-NQO1* cells. Cells were transfected with control siRNA or *CYP1A1* siRNA and cultured in RA condition for 48 h. Total RNA were extracted and subjected to qPCR of *CYP1A1* and the reference gene *OAZ1*. The Ct value of *CYP1A1* was not detected in one group of sample, probably “Ct value > 39”. (n=1-3).
